# Obstacle Avoidance Technique for Mobile Robots at Autonomous Human-Robot Collaborative Warehouse Environments

**DOI:** 10.3390/s25082387

**Published:** 2025-04-09

**Authors:** Lucas C. Sousa, Yago M. R. Silva, Vinícius B. Schettino, Tatiana M. B. Santos, Alessandro R. L. Zachi, Josiel A. Gouvêa, Milena F. Pinto

**Affiliations:** 1 Federal Center for Technological Education Celso Suckow da Fonseca, CEFET, Rio de Janeiro 20271-110, Brazil; lucas.sousa.1@aluno.cefet-rj.br (L.C.S.); josiel.gouvea@cefet-rj.br (J.A.G.); milena.pinto@cefet-rj.br (M.F.P.); 2 Instituto de Engenharia de Sistemas e Computadores, Tecnologia e Ciência, INESC Technology and Science, 4200-465 Porto, Portugal; yago.mrs@gmail.com; 3 Federal Center for Technological Education of Minas Gerais, CEFET-MG, Belo Horizonte 30421-169, Brazil; viniciusbs@cefetmg.br; 4 Science Computer Department, Campus da Praia Vermelha, Federal Fluminense University, Niterói 24210-240, Brazil; tatianambs9@gmail.com

**Keywords:** safety collaboration, human-robot coexistence, robot navigation, shareable workspace, industrial environment

## Abstract

This paper presents an obstacle avoidance technique for a mobile robot in human-robot collaborative (HRC) tasks. The proposed solution uses fuzzy logic rules and a convolutional neural network (CNN) in an integrated approach to detect objects during vehicle movement. The goal is to improve the robot’s navigation autonomously and ensure the safety of people and equipment in dynamic environments. Using this technique, it is possible to provide important references to the robot’s internal control system, guiding it to continuously adjust its velocity and yaw in order to avoid obstacles (humans and moving objects) while following the path planned for its task. The approach aims to improve operational safety without compromising productivity, addressing critical challenges in collaborative robotics. The system was tested in a simulated environment using the Robot Operating System (ROS) and Gazebo to demonstrate the effectiveness of navigation and obstacle avoidance. The results obtained with the application of the proposed technique indicate that the framework allows real-time adaptation and safe interaction between robot and obstacles in complex and changing industrial workspaces.

## 1. Introduction

The increasing adoption of collaborative robots (cobots) in manufacturing environments has significantly transformed industrial production dynamics, enabling closer interaction between humans and robots. The primary challenge in this kind of application is ensuring worker safety without compromising productivity and efficiency [1]. Moreover, integrating sophisticated algorithms and sensors is crucial in augmenting safety measures [2]. In traditional industrial settings, robots and humans are kept apart to maintain safety, achieved through physical barriers and distinct task sequences. However, the scenario of collaborative robotic systems is specifically designed to work alongside human operators and other robots. In this sense, control strategies and sensors are implemented to avoid possible collisions and to ensure safety [3]. Unlike non-collaborative robots, which traditionally operate based on pre-programmed instructions, cobots play an active role in Human-Robot Collaboration (HRC) and Robot-Robot Collaboration (RRC) [4].

Developing an effective navigation approach for autonomous mobile robots or assistive systems while ensuring their safety and security remains a critical challenge in autonomous mobility [5]. Computer vision plays a crucial role in these systems, allowing robots to process and understand visual data from their surroundings for intelligent decision-making [6]. Cameras may be fixed, attached to the robot’s base, or mounted on the end-effector of a robotic arm, commonly known as an eye-in-hand configuration. This configuration provides advantages such as delivering high-resolution point clouds of nearby obstacles and simplifying algorithms by avoiding the need for coordinate transformations, which our research will implement to ensure the highest levels of safety and efficiency in HRC [7].

The camera is also used for mapping, enabling the robot to create detailed, real-time maps crucial for navigating complex environments with varying levels of uncertainty and ensuring that the navigation path is always optimal and safe [8]. Through the visual Self-Locating and Mapping (SLAM) approach with the eye-in-hand camera configuration, our system continuously refines its map as the robot moves through its environment. This dynamic mapping capability ensures that the robot can adjust its path on the fly, avoiding newly detected obstacles and optimizing its route to maintain both safety and efficiency. The high-resolution maps generated through this process are critical for effective HRC, where the safety of human workers depends on the robot’s ability to perceive and respond to its surroundings accurately.

The adoption of cobots in manufacturing environments to assure the safety of human workers without compromising productivity has become of increasing interest as a research topic [4]. As stated in Liu et al. [9], there is no completely safe robot, only secure applications. Hence, many scholars’ studies are based on the capabilities of cobots developed and the safety of their application in manufacturing, and are not related to the robot design itself. Traditional safety measures, such as physical barriers, are inadequate in scenarios where robots and humans must work closely together.

In this sense, this work combines two safety mechanisms, particularly Speed and Separation Monitoring, to allow for dynamic mapping and real-time adaptation to ensure safe navigation and obstacle avoidance in complex environments. This work proposes using the convolutional neural network (CNN) You Only Look Once Version 3 (YOLOv3) algorithm to identify objects in the surroundings. The Tiny version of YOLOv3 was chosen for its lightweight architecture and reduced computational requirements, making it suitable for real-time applications on resource-constrained platforms. Through object detection, the system created a virtual protective boundary with a specified radius around the robot, establishing a safe zone. Incorporating CNN algorithms further augments the Autonomous Guided Vehicle (AGV)’s capabilities by allowing it to discern and interpret the visual data, contributing to the efficiency and adaptability of the AGV’s decision-making process due to its capacity for learning complex patterns and features from visual data [10].

The vision approach employed to support obstacle avoidance, YOLOv3, is based on the speed detection of a single-stage detector like this one. Unlike traditional models like R-CNN and its variants, which use a two-stage process (region proposal followed by classification), YOLO simplifies the task into a single-stage network that simultaneously predicts bounding boxes and class probabilities, making it significantly faster, enhancing detection feedback and achieving better results compared to other models, specifically Region-based CNN (R-CNN) [11], Fast-RCNN [12], and Faster-R-CNN [13]. Furthermore, the YOLOv3 model requires fewer computational resources compared to the newer versions of YOLO, which makes it particularly suitable for environments with hardware constraints; it ensures real-time object detection without the need for extensive hardware upgrades.

Overall, the proposed methodology enables a robot to recognize when it is getting closer to or entering the designated safety area around a human or an object. The depth camera supplies distance data for these detected objects. As the AGV approaches this boundary, a fuzzy reference speed generator is triggered, feeding the robot control system parameters to ensure that the robot moves towards its target securely. All tests were evaluated using a Robotic Operating System (ROS) with Gazebo simulation.

### 1.1. Main Contributions

Due to the increasing need for robust and safe autonomous systems in collaborative environments, this research introduces an approach that combines fuzzy logic with a convolutional neural network (CNN) to enhance robot navigation and obstacle avoidance. This framework integrates visual recognition information into the robot’s autonomous navigation system, thereby ensuring operational safety for both humans and cobots in dynamic workspaces. In order to achieve this, the framework utilizes the YOLOv3 CNN, fine-tuned specifically to detect and identify objects of interest within the robot’s environment. This version was chosen due to its strong compatibility with the ROS ecosystem employed in this work and its suitability for real-time applications on resource-constrained mobile robotic platforms. This visual information is fed into the fuzzy logic module that continuously generates reference speed values for the robot’s internal controller, based on proximity to detected obstacles. The use of fuzzy logic, along with the mapping approach, enables the robot to handle uncertainty and adapt in real-time to guarantee smooth and safe navigation. The contributions of this paper can be summarized as follows:

- Development of a framework based on fuzzy logic capable of integrating the visual recognition information stream from the CNN YOLOv3 algorithm into an autonomous navigation system for greater operational safety for humans and robots.
- The application of a visual-based scene understanding through CNN and mapping approaches towards safe human-robot collaboration.
- Development of a simulated environment and testing of the proposed methodology, demonstrating the technical feasibility of the approach. The proposed strategy is evaluated through simulations in the Gazebo 7.0.0 software and ROS environment [14].

### 1.2. Study Organization

The remaining parts of this paper are organized as follows. Section 2 presents the background of the study and related works, detailing the key safety mechanisms and navigation technologies used in collaborative robotics. This section also highlights the critical challenges addressed in previous studies and positions the proposed approach within the existing literature. Section 3 describes the proposed methodology, including the development of the simulation environment, the fuzzy module, and the autonomous navigation setup. It details the technical setup, algorithms, and the roles of various components in the system architecture. Section 4 discusses the results and performance evaluation of the proposed system, while Section 5 concludes the study and outlines potential future research directions.

## 2. Background and Related Works

A critical component of the HRC scenario is ensuring human safety in the shareable workspace. Adaptable systems offer significant benefits in terms of flexibility and scalability in manufacturing. However, they bring about new and complex types of faults that may arise from human behaviors as well as from the different system components [15]. In this sense, the inherent complexity in designing such safety frameworks presents a significant challenge for HRC.

Many attempts have been made to classify safety control factors in HRC. As stated in Robla et al. [16], the control safety aspects involve psychological and physical issues. Psychological factors concern the human partners’ lack of trust in the robot, while physical factors involve elements that could pose risks to the human partner’s well-being. In both safety control factors, it is necessary to ensure the reliability and predictability of the robot’s actions.

Traditionally, robots have functioned as isolated systems within restricted production environments. However, with the rise of Industry 4.0, there has been a significant shift towards collaborative robotics, emphasizing human–robot interaction (HRI). An in-depth survey on HRC in industrial applications can be found in [17], while a comprehensive review of HRC interfaces and interaction methods is detailed in [18].

Note that the issue of safety in autonomous navigation remains an area with significant gaps [19]. As mobile robots become more prevalent in industrial settings, particularly where they share space with human workers and undertake critical tasks, ensuring their safe operation has become a pressing concern. In the past few years, there has been significant research on mobile robots’ autonomous navigation in industrial settings. Typically, these applications are employed to improve efficiency, reduce operational costs, and ensure safety in daily operational activities, such as material handling [20], inspection [21], and assembly [22]. Different approaches have been proposed for autonomous navigation, including SLAM algorithms [23], sensor fusion techniques [24], and perception systems based on machine learning [24].

As the industrial environment constantly changes, the methods based on pre-defined paths and navigation markers are not well-oriented for safety or efficiency. In order to solve this, research has explored the integration of Light Detection and Ranging (LIDAR) [25], ultrasonic sensors [26], and cameras [27] to improve obstacle detection and avoidance capabilities, as well as the use of deep learning models for object recognition and decision-making in real-time [28].

Several algorithms have been proposed to assist robots in autonomous navigation, allowing robots to adjust their path in the presence of static or moving obstacles [29]. SLAM algorithms enable mobile robots to determine their position while simultaneously creating a map of their environment using sensors such as LIDAR, cameras, and odometry, and have advanced to a level suitable for deployment in AGVs. LIDAR’s collision avoidance capabilities can be leveraged for sophisticated, multi-tiered obstacle avoidance during movement [30]. There are also predictive models being explored that forecast potential collisions by adapting the robot’s speed based on the environment sensing in the robot zone defined based on distance from workers or objects [31,32].

While human–robot collaboration began gaining traction only years after robots were introduced in manufacturing, the issue of robots avoiding obstacles has been a persistent challenge since their early days in the industry. The authors of Ali et al. [33] proposed the use of Fuzzy Logic (FL) to calculate collision risk degree to avoid obstacles for unmanned roads. In [34], the authors applied FL to introduce safety aspects to landing. Zhang et al. [35] introduced a vector field histogram method that builds on the concept of the artificial potential field. The authors of Sun et al. [36] developed a collision avoidance control method using the Dynamic Window Approach (DWA), simultaneously generating feasible dynamic paths and collision avoidance control measures while accounting for potential collision risks. However, neither strategy was tested in the HRC scenario.

The authors of [37] estimated the danger levels in HRC in real-time and generated an alternative robot trajectory when danger exceeded a determined threshold, which combined factors like distance, velocity, and inertia between robot links and humans. A virtual force was used to redirect the robot away from high-risk zones based on relative distance and effective impedance. Subsequent work [38] enhanced this system by incorporating human monitoring, which improved safety through an integrated path planner and motion controller. An interesting approach was proposed in [39]. The authors presented a distributed distance sensor mounted on an industrial robot’s surface as part of the active control strategy. They also designed a collision avoidance control algorithm based around detected humans.

As stated in [40], there is a lack of a holistic consideration of other crucial elements of a working scene, especially when taking a further step towards Proactive HRC. In this sense, the researchers proposed a systematic review of the use of vision-based holistic scene understanding to proactively collaborate in manufacturing environments. They suggested using CNNs for object recognition, human activities, and visual scene understanding. Regarding the use of CNN for obstacle detection, the authors of Alkhaleefah et al. [41] presented a lightweight YOLOv3-mobile network by refining the architecture of YOLOv3-tiny to improve pedestrian detection. In [42], the authors used the YOLOv3 to detect objects in a mobile soccer robot scenario.

These methods primarily operate on 2D image data and lack depth perception, making it difficult to differentiate between low-lying barriers and overhead obstacles. Henke Dos Reis et al. [43] explored a method that integrates YOLO-based object detection with RGB-D images for autonomous navigation, demonstrating that depth information enhances obstacle differentiation. To mitigate this limitation, RGB-D sensors have been integrated into navigation frameworks to provide depth-aware obstacle detection. Studies such as [27,44] demonstrate that combining CNN-based object recognition with depth information significantly improves navigation accuracy. However, most of these works do not explicitly incorporate height-based decision-making, which is crucial for 3D-aware motion planning.

To handle the uncertainty inherent in dynamic environments, FL has been widely explored for adaptive navigation control [45]. Traditional approaches apply fuzzy controllers for local trajectory adjustments, velocity regulation, and collision risk assessment [33]. However, these methods often lack integration with modern deep learning-based perception systems. Lopez-Velásquez et al. [44] proposed a hybrid approach combining deep learning with fuzzy behavior trees to improve autonomous navigation. Their work highlights the benefits of integrating rule-based decision-making with learned object recognition models. Achirei et al. [46] proposed a model-predictive control approach using object detection from CNNs to enhance trajectory planning in logistic environments. However, these methods did not explicitly consider obstacle height and clearance as critical factors in motion planning. Our work builds upon these studies by integrating YOLO for object classification, RGB-D sensors for depth perception, and fuzzy logic for adaptive control. This combination allows for more accurate obstacle classification and safer trajectory adjustments.

Table 1 categorizes each approach based on its type, adaptability, computational complexity, real-time applicability, and suitability, specifically within HRC contexts. This comparative framework allows for a clearer understanding of the strengths and limitations of each method, highlighting how different techniques address the critical challenges in collaborative robotics. Note that advanced approaches like real-time danger estimation and sensor-based methods provide higher adaptability and suitability for HRC but come at the cost of increased computational demand.

As can be seen, using CNNs, like YOLO, further improves detection and avoidance capabilities by recognizing objects in complex settings, allowing for dynamic path adjustments. This technique, along with other technologies, such as RGB-D cameras, robust control algorithms, and costmap functions, such as is the proposition of this work, can ensure reliable and adaptive navigation in constantly changing industrial environments. It is possible to verify that the proposed approach distinguishes itself through the use of SLAM combined with Fuzzy Logic and RGB-D cameras integrated with YOLOv3 CNN for dynamic obstacle detection and avoidance. Our proposed work incorporates navigation strategies and safety measures to enhance operational safety in human–robot interactions in complex and dynamic environments.

## 3. Proposed Methodology

**Figure 1:**
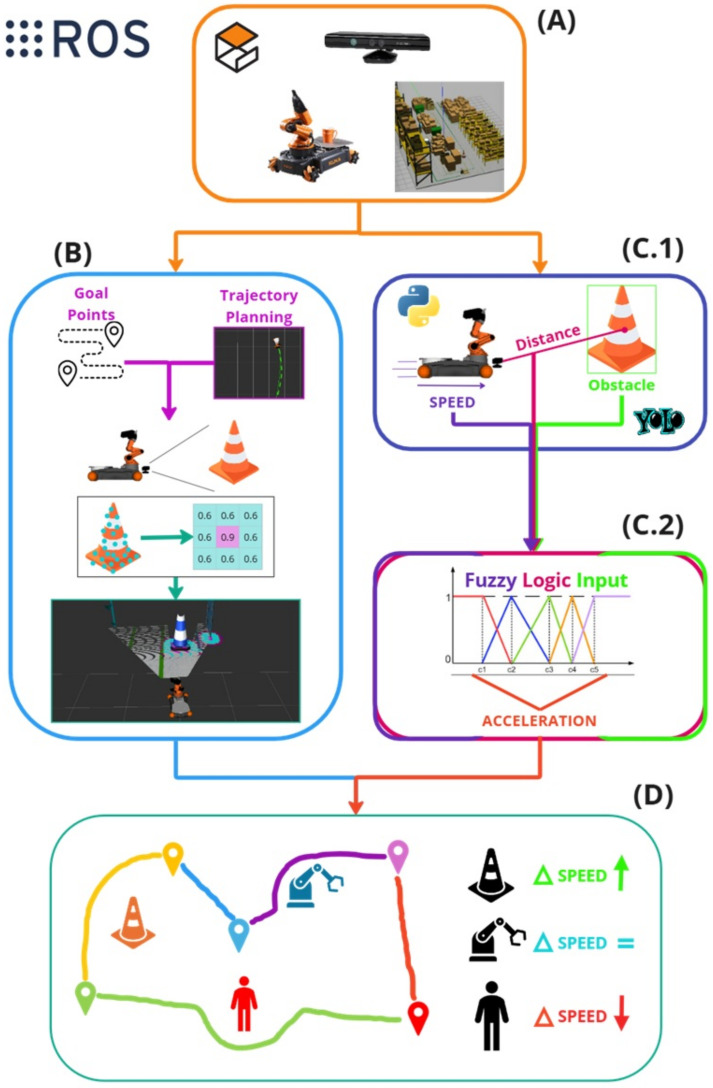
Overview of the proposed method. The target application scenario is a warehouse with a mobile platform equipped with a camera (**A**). This setup enables the implementation of trajectory planning methods that utilize goal point coordinates. The system enables RGB-D odometry-based path planning by leveraging the Kinect Camera to create a costmap from the depth camera’s point cloud (**B**). In parallel, the method employs YOLOv3 for object detection to identify specific obstacles in the path. The solution uses the depth camera to measure the distance to the centroid of detected objects while also gathering speed data from the robot’s sensors (**C.1**). This information is input into the fuzzy logic module (**C.2**), where it is processed. Based on the object class, robot speed, and distance, the module adjusts the robot’s reference velocity while maintaining its trajectory (**D**).

The proposed architecture for an autonomous robotic system is designed for a collaborative environment. The system operates in a simulated 3D environment with typical warehouse objects such as cones, human workers, and robots. The chosen robot is a KUKA Youbot, equipped with a Kinect RGB-D camera on its end-effector to capture color and depth information, as shown in Figure 1A. The processing pipeline is divided into several key stages. In stage (B), the robot determines its movement goals and plans a trajectory to reach those points while avoiding obstacles. Real-time spatial mapping and goal point identification assist in the trajectory planning stage, helping the robot define safe paths. Next, the robot’s sensory inputs are processed in parallel for two main tasks: object detection and trajectory adjustment. In the object detection module (Figure 1(C.1)), the system uses distance data along with the YOLO algorithm to identify nearby obstacles. This is further explained in Section 3.3. The robot’s internal speed controller is fed by the fuzzy logic-based mechanism (Figure 1(C.2)), which continuously generates velocity reference values based on detected distances, ensuring smooth and efficient movement. The details are explained in Section 3.4. Finally, the robot’s integrated navigation system (details can be found in Section 3.5), as seen in Figure 1D, combines data from previous modules to adjust its speed and trajectory continuously, allowing it to move safely through dynamic environments by maintaining an optimal speed and avoiding potential collisions.

### 3.1. Simulation Setup

The test computer setup includes an Intel i7-11800H processor, 16GB DDR4 memory, and an NVIDIA GeForce RTX 3050 Ti GPU with 12GB VRAM. The system runs on Ubuntu 16.04 and uses the Gazebo AWS RoboMaker Small Warehouse World [47], which features boxes, trash bins, pallets, and other elements to challenge obstacle avoidance tests. All computer vision and machine learning algorithms are based on OpenCV 3.4.5 and TensorFlow 1.4. It is important to note that the core packages and functionalities of ROS, including those utilized in this study, remain supported and compatible with more recent Ubuntu versions (e.g., 20.04 or 22.04) via updated ROS distributions like ROS Noetic or ROS 2.

**Figure 2:**
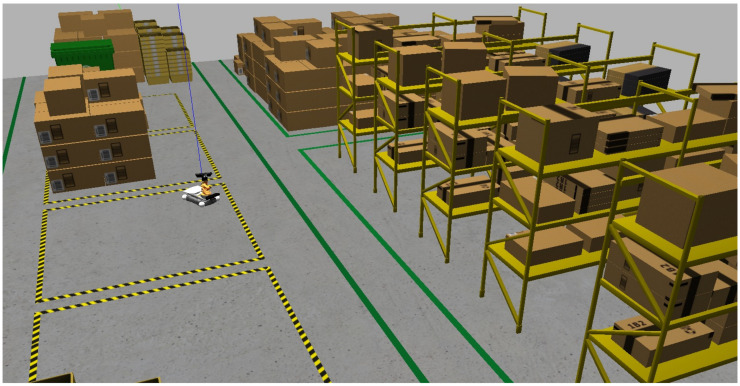
Warehouse scenario and KUKA Youbot robot in the Gazebo software.

The robot selected for the project is KUKA Youbot, for which the robot model, drivers, and controller are available on GitHub [48]. The proposed study can be accessed via the following link (https://github.com/LucasSousaENG/lucas_masters, accessed on 1 April 2025). Figure 2 presents the simulation environment and the KUKA Youbot robot with a kinetic RGB-D camera added by the authors.

Once the simulation environment is initialized, a launch file in ROS deploys the KUKA Youbot. A Kinect camera is also integrated into the setup and mounted on the robot’s base frame (i.e., eye-in-hand camera). This camera is calibrated to capture both standard RGB images and depth information, which is essential for navigation and obstacle-avoidance sensor-based tasks. The system activates the RViz node for robot visualization with these launch files.

### 3.2. Autonomous Navigation

The navigation system proposed in this work is designed for full 3D motion planning, integrating RGB-D camera data to generate a comprehensive three-dimensional environment representation. Unlike conventional 2D costmaps, which only consider a planar representation of obstacles, our system employs a dense PointCloud2 representation from the Kinect camera to construct a 3D obstacle map. This ensures that both ground-level and aerial obstacles are considered, improving safety and efficiency in autonomous navigation.

**Figure 3:**
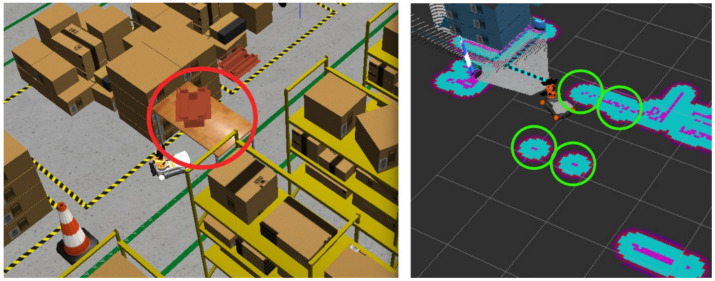
A suspended toolbox (red circle) placed on a table in front of the robot’s path during the navigation phase. Note that while the table itself and the object above it are detected, they are not marked as obstacles in the costmap. However, the table’s legs are correctly identified and registered as obstacles (green circles).

The costmap is generated dynamically using real-time sensor data, incorporating depth information from the /camera/depth/points topic. This PointCloud2 data enables the system to accurately interpret the spatial structure of the environment, including obstacle height, free space beneath suspended objects (as illustrated in Figure 3), and dynamic changes over time. This capability is critical to allowing the robot to adapt its trajectory in a three-dimensional space rather than being limited to a strictly planar path.

**Figure 4:**
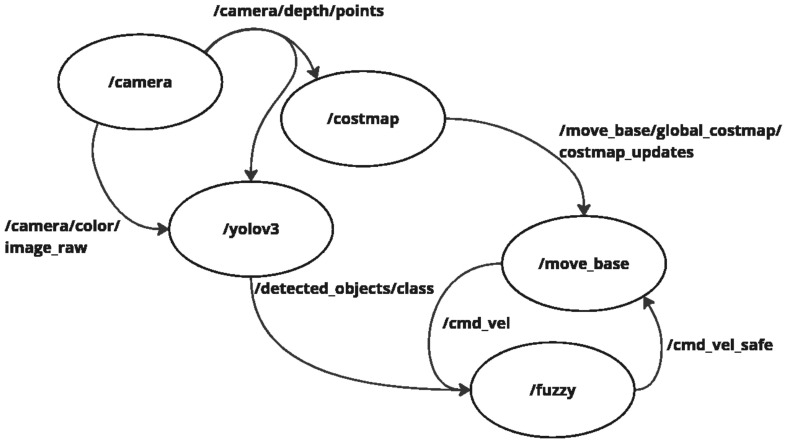
Active ROS nodes and topics.

The navigation stack is based on the ROS framework and is illustrated in Figure 4. The *move_base* node integrates global and local planners to ensure smooth, obstacle-free navigation. The global planner defines an initial path from the robot’s start position to the goal, using a 3D-aware costmap that considers ground and elevated obstacles. The local planner continuously adjusts the trajectory in real time based on sensor feedback, ensuring that obstacles detected at different heights influence the motion strategy.

Basically, in autonomous navigation, the camera is the primary sensor, providing depth and color images. The depth information from the topic */camera/depth/points* serves as the primary input for the */costmap* node, which constructs a map [49]. This allows the system to identify obstacles and an inflation layer defining a general safety buffer around detected obstacles.

However, not all obstacles are treated equally. The color image from the topic */camera/color/image_raw* is processed by the YOLOv3 node, but not for general obstacle detection. Instead, YOLO is specifically used to classify certain objects requiring a differentiated safety zone, such as humans or fragile warehouse equipment. When such objects are detected, a customized safety radius is applied (previously proposed by the authors in [50]), ensuring a greater clearance area in their vicinity. This distinction enhances the robot’s ability to interact safely within human-robot collaborative environments.

The *move_base* node plays a central role in trajectory planning to ensure safe and efficient navigation. The global planner determines the primary path from the starting position to the goal, leveraging a pre-existing environment map to avoid static obstacles. Meanwhile, the local planner continuously adjusts the trajectory in real-time based on sensor feedback, allowing the robot to dynamically avoid moving obstacles and ensure smooth navigation [51].

The interaction between these planners is managed by the costmap, updated via the topic */move_base/global_costmap/costmap_updates*. Additionally, the *move_base* node generates velocity commands (*/cmd_vel*) based on real-time odometry and depth data from the RGB-D camera. The fuzzy logic module further processes these velocity references, which applies dynamic adjustments based on obstacle classification and safety zone adaptation, resulting in the */cmd_vel_safe* topic [52]. This ensures that the system avoids collisions and adjusts speeds and behaviors according to the type of obstacle encountered.

The decision to rely mainly on odometry and camera-based inputs was made to create an adaptable, cost-effective solution with minimal computational demands. This design maintains simplicity while allowing flexibility to integrate additional sensors, such as LiDAR [25], to improve SLAM-based navigation [53], further enhancing the robot’s autonomous capabilities. This modular approach ensures the system remains scalable for more complex industrial environments.

To guarantee an optimized configuration, some parameters are fine-tuned, which include the robot’s height and width to account for its physical dimensions, filtering out the ground to prevent it from being wrongly classified as an obstacle, and setting the inflation radius around detected obstacles to maintain safe clearance. Table 2 presents the set of parameters used [54].

Finally, to automate the navigation process, which involves defining the target coordinates relative to the robot, the authors decided to develop an algorithm that reads target locations from a YAML file and publishes the setpoints to the */move_base_simple/goal* topic via the *publish_goals* node. The script subscribes to the odometry topic to continuously monitor the robot’s position and orientation. As the robot follows the path, the algorithm compares the current position over the predefined tolerance of the goal. Reaching this threshold, the script advances to the next target point, repeating the process until the robot arrives at the goal position.

### 3.3. Object Detection

This study employs version 3 of the real-time object detection algorithm You Only Look Once (YOLO), a deep learning algorithm for object recognition. It belongs to the category of single-shot detectors, which divides the image as a grid and simultaneously predicts multiple bounding boxes and their corresponding confidence scores and class probabilities, significantly increasing the speed of the object detection [55]. This work uses YOLOv3-Tiny as a part of the framework due to its balance between computational efficiency and real-time performance, which is important for onboard processing on resource-constrained mobile robots like the KUKA Youbot. While newer models such as YOLOv5 and Faster R-CNN offer improved accuracy in object detection tasks, they typically require significantly more computational power and memory, which can compromise real-time responsiveness when deployed in embedded or low-power systems. In contrast, YOLOv3-Tiny provides sufficiently accurate results while ensuring fast inference, making it more suitable for robotic applications where responsiveness is critical.

**Figure 5:**
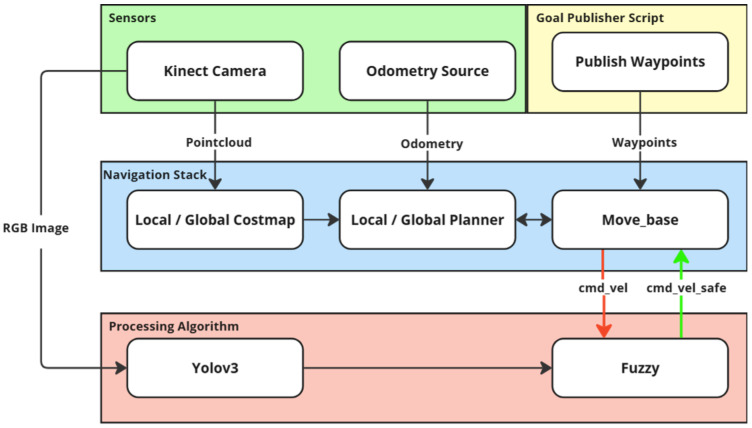
Architecture based on the ROS navigation stack and adapted to use object identification in order to provide safer autonomous movement.

Currently, the system relies on a Kinect RGB-D camera for both color and depth information, as shown in Figure 5. However, the approach can be extended to integrate additional sensor modalities, such as LiDAR, stereo vision systems, or ultrasonic sensors, depending on the available hardware. This flexibility enables the framework to adapt to a wide range of industrial scenarios, further enhancing its robustness and generalization capability in dynamic environments.

**Figure 6:**
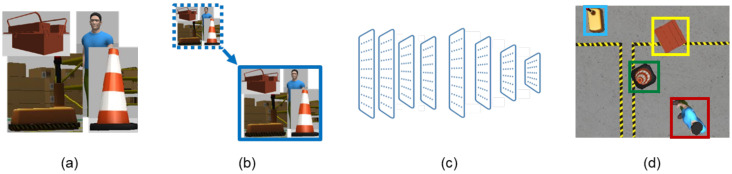
YOLOv3 simplified structure. (**a**) Input image. (**b**) Image resizing. (**c**) Convolutional layers. (**d**) Object detection.

The YOLOv3 architecture uses Darknet-53 as the model backbone, which is composed of 53 layers based on residual networks (ResNets) and a series of convolutional layers with batch normalization and leaky ReLU activation functions. This design allows highly efficient hierarchical feature extraction from the input image, capturing low-level (e.g., edges and textures) and high-level details (e.g., object parts and semantics). In the main framework, YOLOv3 makes predictions in a multi-scale approach, in which the three scales are typically 52 × 52, 26 × 26, and 13 × 13 grid sizes, corresponding to detecting large, medium, and small objects. This multi-scale approach, combined with the efficient feature extraction by Darknet-53, makes YOLOv3 highly effective for real-time object detection in complex environments, such as industry workshops and warehouses [56]. Figure 6 illustrates a YOLOv3 simplified structure.

To improve mobile robot navigation and safety in a collaborative warehouse environment, the authors have created a custom YOLO dataset with 250 images to identify people, toolboxes, cones, and warehouse robots. These classes were selected to represent common elements of visual awareness in such environments.

- People: As the most critical class, ensuring the safety of humans is preeminent. The robot must prioritize avoiding collisions with people to maintain a safe collaborative space.
- Toolboxes: Representing static objects that pose no immediate danger, toolboxes allow the robot to maintain higher speeds and require less braking mid-course.
- Cones: Normally viewed as markers for area delimitation, cones require exponential braking as the robot approaches to ensure that it respects designated boundaries.
- Warehouse Robots: As dynamic objects, other robots require careful navigation to avoid collisions, prioritizing balancing speed and caution during the route.

By training the neural network in these classes, we aim to create a system that optimizes efficiency and safety in a collaborative warehouse setting. The dataset has synthetic images from the Gazebo simulator environment, labeled as **person**, **toolboxes**, **cones**, and **warehouse robots** using the LabelImg [57] annotation tool. During training, the YOLOv3 configuration uses a transfer learning technique to train the model on this small dataset. In order to mitigate the limitations of using a relatively small dataset, we employed a set of data augmentation strategies (i.e., flipping, scaling, rotations, and brightness) to improve generalization and simulate real-world variability. Partially occluded objects were also included to reflect the dynamics of collaborative warehouse environments. Table 3 summarizes all the parameters used.

**Figure 7:**
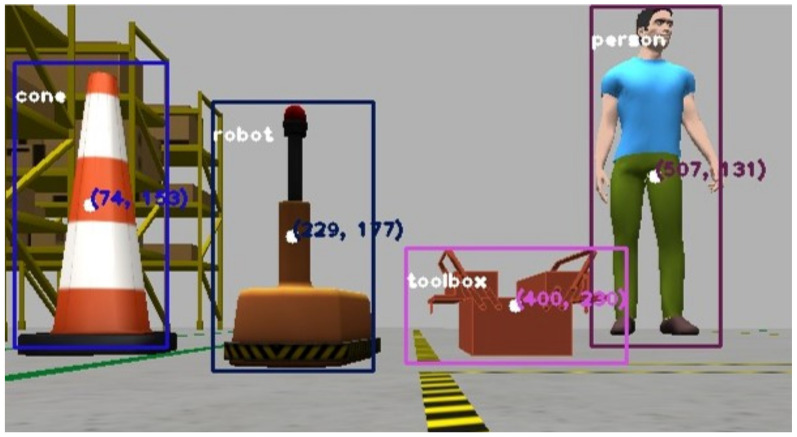
YOLO model detection and centroid estimation.

The proposed algorithm (see Algorithm 1) calculates a region of interest (ROI) by taking the median of the YOLOv3 detection coordinates and creating an array of the nearest depth points at the center of this ROI. The script processes the nearest point, extracting the coordinates and distance, which are used as the primary distance measure from the robot camera to the detected object. The coordinates, classification, and object distance are then published as ROS topics. This data is integrated into the robot’s navigation and control systems, with a fuzzy logic module that continuously provides velocity and trajectory references based on the input image and YOLOv3 prediction. Figure 7 illustrates the moving base close to the detected classes.
**Algorithm 1** Autonomous Collaborative Environments Obstacle Avoidance Algorithm 1:$x_{0}$**,**$y_{0}$: Pixel coordinates centroid 2:ROI: Region of interest 3:*u***,***v*: Pixel coordinates of nearest point to the center 4:fx**,**fy: Focal lengths in x, y-axis 5:cx**,**cy: Optical center coordinates 6:$D_{o}$: Distance from detected object 7:$V_{i}$**,**$V_{o}$: Input and Output Velocity from Fuzzy process 8:**$f_{a}$**: Multiplicative factor separated by object class 9:**$V_{of}$**: Final velocity10:Initialize YOLOv3 model and load class labels11:Initialize Point Cloud data distance estimation12:Initialize Fuzzy Logic Speed module13:**while** Running **do**14:    // Parallel Block Begins15:    **fork**:16:      [Process 1] YOLOv3 obstacle detection17:        Gets /camera/color/image_raw18:        Applies to resize and blob creation19:        Extract bounding box20:        Calculate the centroid of the bounding box21:        $x_{0},y_{0}\leftarrow \mathrm{bounding}\;\mathrm{box}\;\mathrm{centroid}$22:      [Process 2] Obstacle distance estimation23:        Gets /camera/depth/points24:        $ROI=(x_{0},y_{0})+-Threshold$25:        Extract (x,y,z) for each point in ROI26:        Calculate distance from the camera to point:(1)$$\;u=\left(\frac{x\cdot fx}{z}\right)+cx,\;v=\left(\frac{y\cdot fy}{z}\right)+cy$$27:        **if** u,v coordinates are nearest to $\left(x_{0},y_{0}\right)$28:          **z** = Object distance29:        /fuzzy_input←[u,v],Classlabel30:        Publish /fuzzy_input31:      [Process 3] Fuzzy module32:        Gets /fuzzy_input33:        Append /fuzzy_input as $D_{i}$ and (class)34:        Append /cmd_vel as $V_{i}$35:        // General Fuzzy Logic Rules36:        Apply Acceleration Rule based on $D_{i}$, $V_{i}$37:        // Class-based adjustment function38:        $\mathrm{Multiplicative}\;\mathrm{Factor}\;f_{a}\leftarrow \mathrm{Value}\;\mathrm{from}\;\mathrm{Table}\;6\;$39:        $V_{of}\leftarrow V_{o}\times f_{a}$40:        $/\mathrm{cmd}\_\mathrm{vel}\_\mathrm{safe}\leftarrow V_{o}$41:        Publish/cmd_vel_safe42:      **join**43:    // Parallel Block Ends44:**end while**

### 3.4. Fuzzy Logic Processing

**Figure 8:**
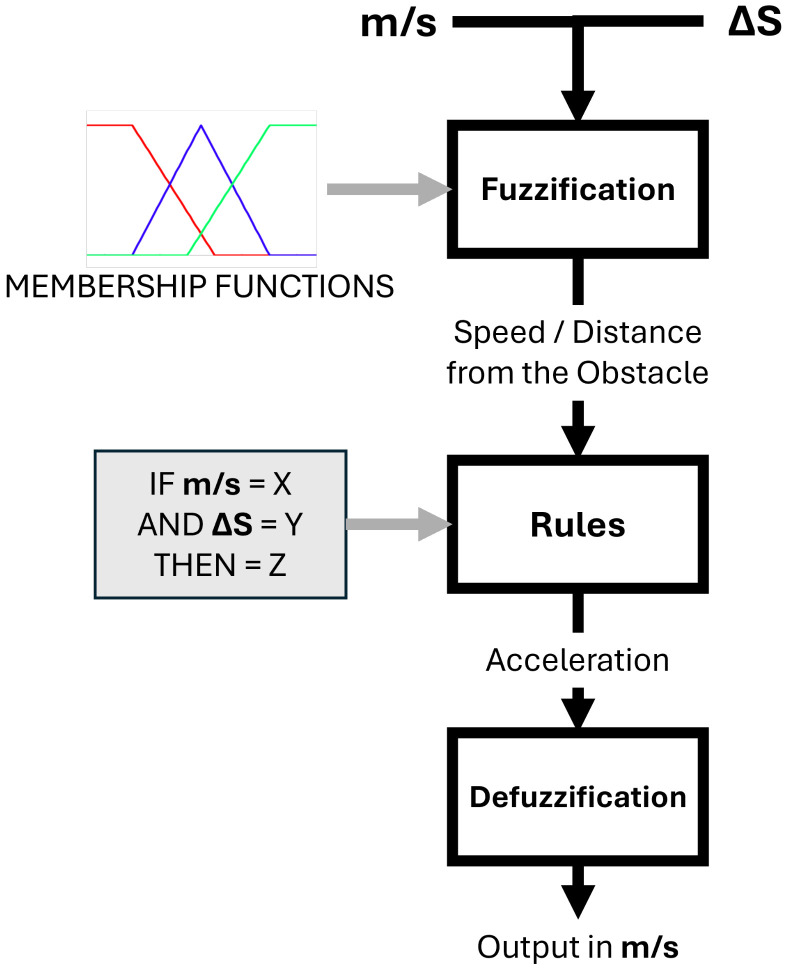
Fuzzy flowchart implementation in this study. The diagram illustrates the process where precise inputs, such as speed (m/s) and distance (ΔS), are fuzzified into degrees of membership. Fuzzy rules are then applied to determine acceleration, and defuzzification converts the fuzzy output into a precise speed value (m/s).

Fuzzy logic uses the human capability to interpret partial truths, operating on a continuous scale between true and false. It quantifies how closely an input aligns with a specific result, which is appropriate for handling uncertainty and imprecision [58]. This kind of controller is widely used to control different processes, especially to ensure and improve system robustness. This makes it an ideal choice for projects like this, where the results are based on the truth values spectrum, and the robot’s acceleration (faster or slower) is based on the proximity of obstacles. In this context, fuzzy-based controllers are well recognized for their ability to improve both robustness and adaptability by dynamically tuning the controller parameters in system outputs [59]. Figure 8 explains each step of the fuzzy logic implementation in the project in parallel with input types and variables.

The fuzzy inference system operates based on the standard Mamdani inference approach [60], employing the defuzzification centroid to determine the output. Table 4 presents the fuzzy rules of the robot’s speed. To define the magnitude of the angular velocity, this table describes the “Turn” (Turn rate) intensity: “H” and “L” refer to high and low, and “VH” and “VL” stand for very high and very low.

The fuzzy module implemented in this work also defines the robot’s base acceleration. The fuzzy rules used for providing acceleration references consider the current velocity and the distance to obstacles. Based on the measured variables, the module calculates the appropriate reference values for acceleration commands to the robot. The acceleration is influenced by “distance” (distance to object) and “velocity”, which regards velocity to object, as presented in Table 5.

The fuzzy implementation of this work can be divided into three main stages, which are fuzzification, rule evaluation, and defuzzification. **Fuzzification** involves transforming precise (CRISP) input values into degrees of membership within a fuzzy set. In the current work, this stage applies to the velocity, the distance, and the class data, collected in the trained network model. These CRISP inputs are mapped to determine the truth degree, indicating how closely the variables fit within specific groups, while the distance is measured based on the obstacle’s proximity to the camera. Finally, the velocity is based on the Kuka Youbot’s default speed, for which zero indicates stopped, and one is the maximum speed. The class value represents the object type.

Meanwhile, the **rule evaluation** phase can be presumed as an “IF-THEN” statement, which is applied to each fuzzified input. A corresponding set of rules is generated that defines the system’s response. This step classifies both distance and velocity groups to produce an arbitrary danger zone, which will be the input of the next stage.

**Figure 9:**
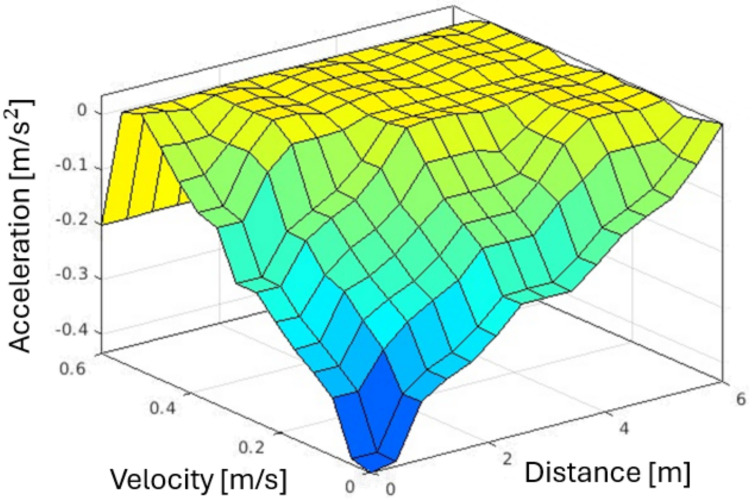
Fuzzification process surface plot.

**Figure 10:**
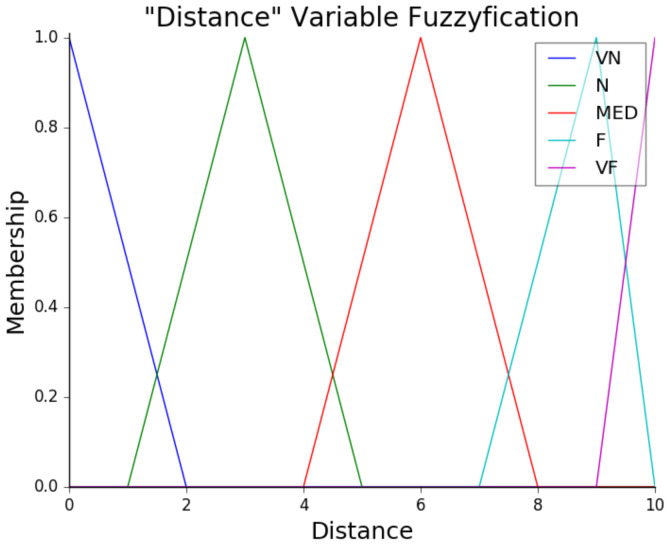
Membership function of the distance.

Conversely, **defuzzification** receives the danger zone value and translates it into a precise value. This involves converting acceleration and deceleration rates into speed adjustments for the robot. Then, the resulting output is modified by a class magnitude factor, which adjusts the acceleration based on the type of obstacle, e.g., if the robot detects a human, it reduces the speed more significantly than it would when encountering a toolbox or cone. Figure 9 presents the fuzzification process surface and Figure 10 the membership function of the distance.

Table 6 defines some arbitrary acceleration multiplier factors. These values are chosen based on the hazard of a collision if any of the classes are detected in the robot’s path. The authors consider both static (e.g., cone or toolbox) and dynamic issues (e.g., a person or robot). Based on this framework for the fuzzy module, the robot can dynamically navigate its environment by continuously adjusting its speed to avoid obstacles and optimize its trajectory. This real-time object detection system with a fuzzy module is key in ensuring the robot reaches its target destination safely.

### 3.5. Dynamic Environment

**Figure 11:**
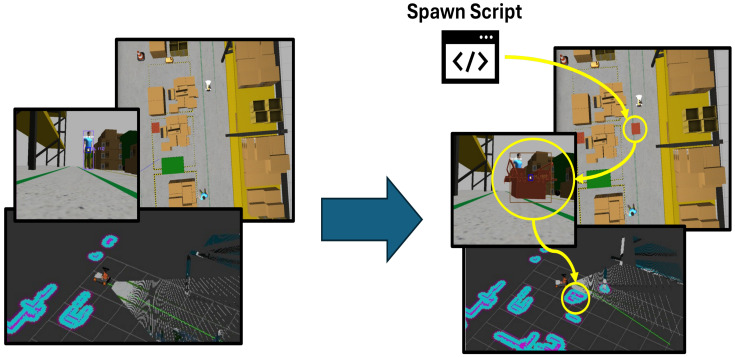
Object spawn based on robot`s position to simulate a sudden object placement in the trajectory course.

In a busy workplace, the environment is rarely static. People are constantly moving objects, creating unexpected obstacles in the path of collaborative robots, which can lead to sudden changes in their trajectories and potentially put nearby workers at risk. Considering these real-world scenarios, a static test environment often does not accurately represent workplace dynamics. This section, therefore, focuses on simulating sudden changes in the environment by introducing obstacles that appear unexpectedly during trajectory testing. Figure 11 represents the idea implemented in the project.

To test real collaborative incident-prone work environments, an idea to pop up an obstacle in front of the robot’s path was developed in a script format that reads the robot’s odometry in real-time. When the robot reaches a specific point, the script places an object in its path, mimicking situations like a dropped toolbox or a suddenly appearing obstacle. This setup tests how effectively the robot’s algorithm adapts to unexpected events, ensuring safety and adaptability in a real-world environment.

**Figure 12:**
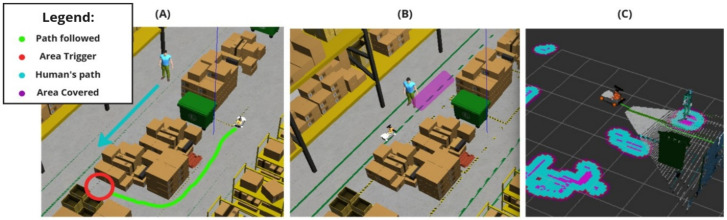
(**A**) Starting test phase describing the steps and actions of each part in the moving person’s script. (**B**) Area covered by the human during the testing phase following the moment it is detected by the robot. (**C**) Costmap created showing human detection by the depth camera and the costmap trail placed by its moving motion.

To further evaluate the system’s performance in a truly dynamic collaborative environment, an additional test was conducted involving a moving human obstacle. In this test, when the robot passes a specific trigger spot, a human obstacle begins moving along the negative Y-axis (toward the robot). This scenario mimics a dynamic collaborative environment where a person moves unpredictably, requiring the navigation stack to continuously recalculate the path while ensuring safety. The proposed algorithm successfully braked the robot to avoid collisions and maintained the safest possible distance from the moving human. This test highlights the system’s ability to adapt to dynamic environments while prioritizing safety and efficiency. Figure 12 explains the algorithm functionality.

### 3.6. System Compatibility and Sensor Flexibility

The proposed implementation is mostly independent of both hardware and ROS versions, as it relies exclusively on standard ROS interfaces that remain consistent across platforms and versions. The system requires only three universal components: provision of sensor data in standard PointCloud2/Image formats and command velocity input in the standard Twist format. This design ensures compatibility with any robotic platform that meets these interface requirements.

For multi-sensor configurations (e.g., separate RGB camera for detection and depth sensor for navigation), the algorithm requires only minor adjustments to the bounding-box-to-point-cloud correlation method. Specifically, the coordinate transformation between sensors is handled automatically through ROS tf2, with any necessary resolution scaling or projection adjustments being performed during the detection-to-navigation data fusion step. This ensures consistent spatial referencing regardless of sensor combinations, provided proper calibration is maintained. The only potential constraints would come from hardware-specific factors such as computational resources or sensor driver availability, rather than from our implementation’s architecture.

Currently, the system relies on a Kinect RGB-D camera for both color and depth information, as shown in Figure 5.However, the approach can be extended to integrate additional sensor modalities, such as LiDAR, stereo vision systems, or ultrasonic sensors, depending on the available hardware. This flexibility enables the framework to adapt to a wide range of industrial scenarios, further enhancing its robustness and generalization capability in dynamic environments.

## 4. Results and Discussion

### 4.1. Neural Network Performance

**Figure 13:**
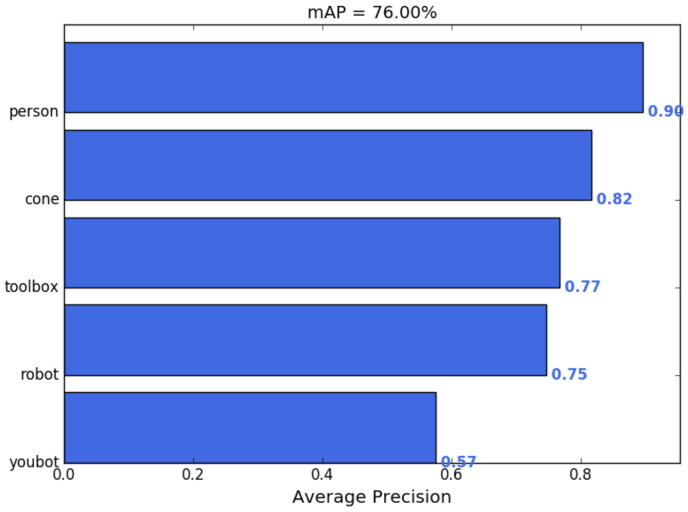
The mAP values corresponding to the trained YOLOv3 Custom Model.

The trained YOLOv3 model effectively detected key objects during the environment tests, prioritizing the accuracy of the person class for safety. The model was trained on a custom dataset of 250 images using data augmentation and transfer learning. The model achieved a mean average precision (mAP) of 76.00% across five classes: cone, person, robot, toolbox, and Youbot. mAP is a commonly used evaluation metric in object detection that reflects both the accuracy and localization quality of predicted bounding boxes. Specifically, mAP represents the mean of the average precision (AP) computed across all object classes, where AP accounts for both precision (how many of the detected objects were correct) and recall (how many of the actual objects were detected). A high AP (e.g., 89.60% for the person class) does not imply that 10.4% of persons were entirely missed, but rather that, across varying confidence thresholds, there was a 10.4% discrepancy in achieving perfect precision and recall. Moreover, part of this discrepancy may be due to minor inaccuracies in bounding box localization or confidence score variations, rather than complete detection failures. Figure 13 provides a visualization of the mAP across all classes and showcases the system’s ability to balance accurate detection and efficient navigation. Furthermore, class-specific adjustments during training should enhance both the robustness and reliability of the detection system.

The person class achieved the highest average precision (AP) at 89.60%, followed by the cone (81.57%) and toolbox (76.69%) classes. These results reflect the training’s emphasis on safety-critical classes, where high precision (93%) and recall (94%) for the person class ensure consistent and reliable detection. Note that the Youbot class displayed the lowest AP at 57.47%. However, it is important to observe that this class was not the primary focus of the training process. The Youbot, being the robot utilized in the project, was not expected to exhibit the same detection accuracy as other common warehouse robots, which were represented by the “robot” class in the training dataset. Therefore, the detection performance for the Youbot should not be seen as a theoretical limitation but rather as an intended outcome, as its detection was less critical compared to other object classes.

**Figure 14:**
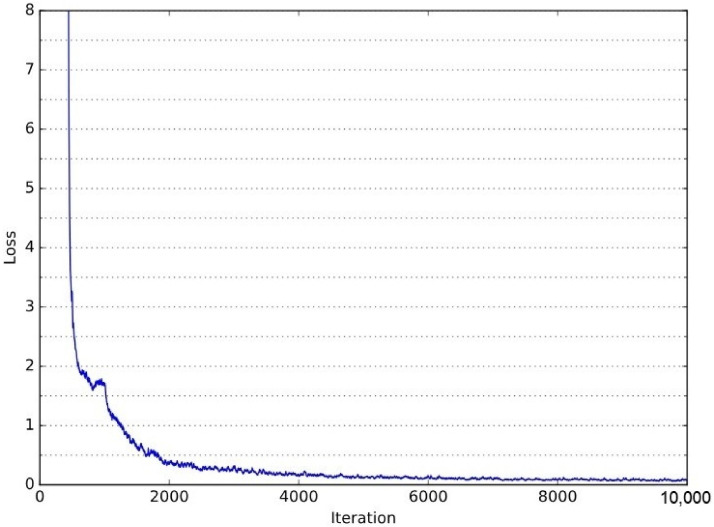
Evolution of the validation loss during the YOLOv3 training. The steady decrease and stabilization of the curve indicate effective learning and convergence.

Figure 14 presents the validation loss curve throughout the YOLOv3 training process. The loss decreases significantly in the initial iterations, demonstrating rapid learning, and progressively stabilizes as training advances. This behavior indicates effective parameter optimization and convergence. The corresponding training Colab notebook, detailing the entire process, is available at the following link: YOLOv3 Training Notebook (https://github.com/LucasSousaENG/lucas_masters/blob/master/yolov3_lossmethod.ipynb, acessed on 1 April 2025).

### 4.2. Autonomous Collaborative Results

**Figure 15:**
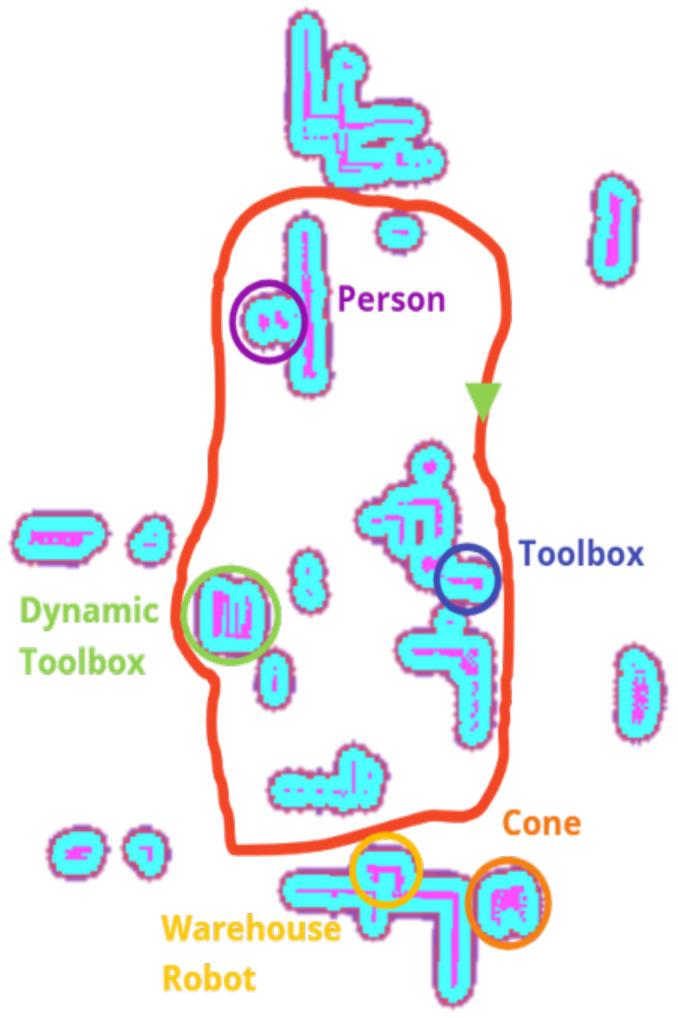
A real-time costmap generated by the robot’s Kinect RGB-D camera with objects, highlighting objects, the start of the test, and the average path.

This section presents the results of the proposed autonomous collaborative solution’s performance in a warehouse environment. Figure 15 illustrates the map generated by the robot. This costmap effectively identifies potential obstacles and available spaces within the workspace. Furthermore, the continuous map updating enables the robot to maintain a high level of spatial awareness, which is crucial to guarantee smooth and efficient operations in collaborative scenarios.

**Figure 16:**
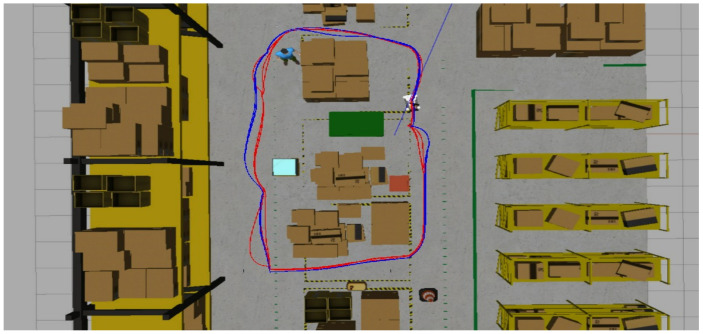
Comparison of navigation paths in a dynamic warehouse environment. The blue trajectory corresponds to the “Default” configuration (standard ROS Navigation Stack without semantic perception), while the red trajectory represents the proposed “Solution”, which integrates object detection (YOLOv4), depth sensing (RGB-D), and fuzzy logic-based adaptive control.

Figure 16 compares 2D paths derived from odometry data, highlighting the effectiveness of different situations. The red and blue lines show the robot’s trajectories under different configurations, demonstrating the system’s effectiveness in obstacle avoidance and the effectiveness of object-aware navigation using Fuzzy Logic in simulated environments. In this context, the “Default” configuration refers to the standard ROS Navigation Stack setup, which performs 2D costmap-based path planning and obstacle avoidance without any semantic perception or adaptive control mechanisms. On the other hand, the “Solution” corresponds to our proposed approach, which integrates object classification through YOLOv3, depth perception via Kinect RGB-D sensing, and a fuzzy logic controller that dynamically adjusts the robot’s navigation strategy based on the type and proximity of detected obstacles.

After conducting several tests comparing the standard navigation stack and the proposed method for an autonomous obstacle-avoiding mobile robot in human-robot collaborative warehouse environments, the robustness of the algorithm has been demonstrated, particularly in settings where safety and adaptability are the biggest concerns. Table 7 summarizes the results of these experiments, indicating that this approach not only enhances safety but also improves travel efficiency.

The data show that the “Solution” achieved faster average lap times than the “Default” setup in static, sudden toolbox appearance, and dynamic tests (moving person). The braking logic, based on object proximity, provides smoother speed control, leading to quicker and more consistent laps. Controlled deceleration minimizes the need for sharp turns or abrupt corrections, allowing the path-planning algorithm to function with less strain and deliver a more consistent route. This also enhances the robot’s cargo transportation capability, reducing the chance of an overturn in the load.

The outcomes also show that path variation metrics indicate a slightly smoother trajectory with the “Solution”, especially in static environments. In sudden tests, where a toolbox unexpectedly appeared in front of the robot during the track, the “Solution” effectively balanced speed and safety, maintaining collision-free performance. Similarly, in dynamic tests involving a moving human obstacle, the “Solution” demonstrated superior adaptability and safety margins, as evidenced by the larger distances maintained compared to the default navigation stack. This improvement in trajectory smoothness and safety confirms that the vision-fuzzy system offers a more robust and adaptive solution than traditional odometer-based navigation.

### 4.3. Safety Conduct Analysis

Safety is the biggest concern in autonomous robots operating in collaborative environments, as real-world applications involve dynamic and constantly changing scenarios. To evaluate the safety performance of the proposed system, the authors conducted both static and dynamic tests. During these tests, odometry data were collected as the robot navigated through environments with obstacles, providing distance measurements from detected objects. These data were crucial for assessing the effectiveness of the proposed system in maintaining a larger safety buffer without significantly compromising task efficiency.

**Figure 17:**
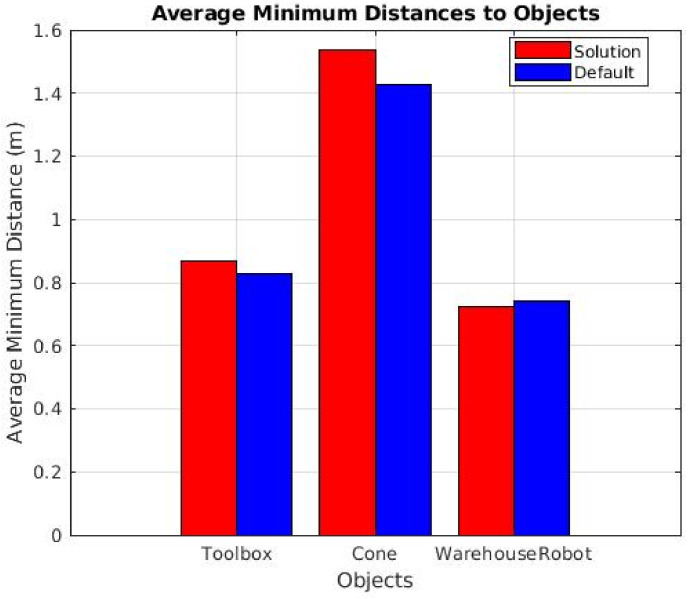
Minimum distances from the robot to the respective objects. Data were acquired during static toolbox tests.

**Figure 18:**
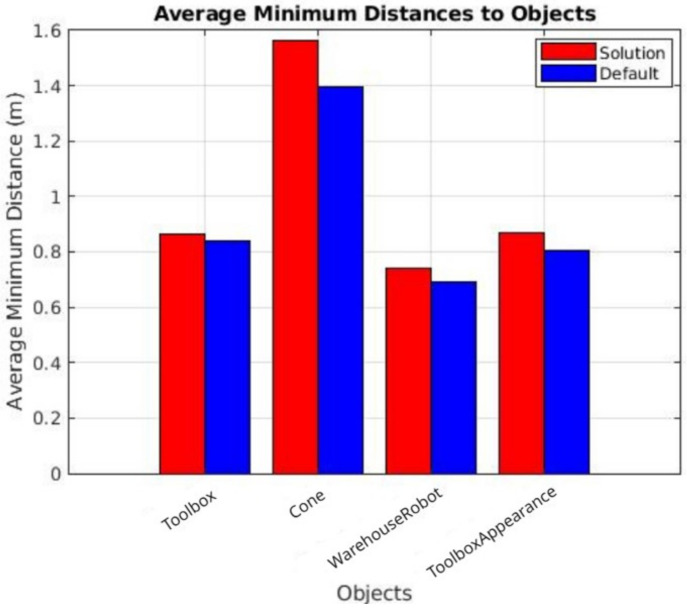
Average minimal distances from the robot to the objects. Data were acquired during sudden toolbox appearance tests.

Figure 17 and Figure 18 demonstrate that, in nearly all instances, our solution maintained a greater distance from detected objects compared to the default navigation method. These results highlight the ability of our approach to allow the robot to operate with a larger error margin while ensuring a safe distance from obstacles. Notably, during the sudden appearance toolbox tests, the average minimum distance consistently increased across all trials, underscoring the effectiveness of our object-aware navigation system in enhancing safety without sacrificing operational efficiency.

**Figure 19:**
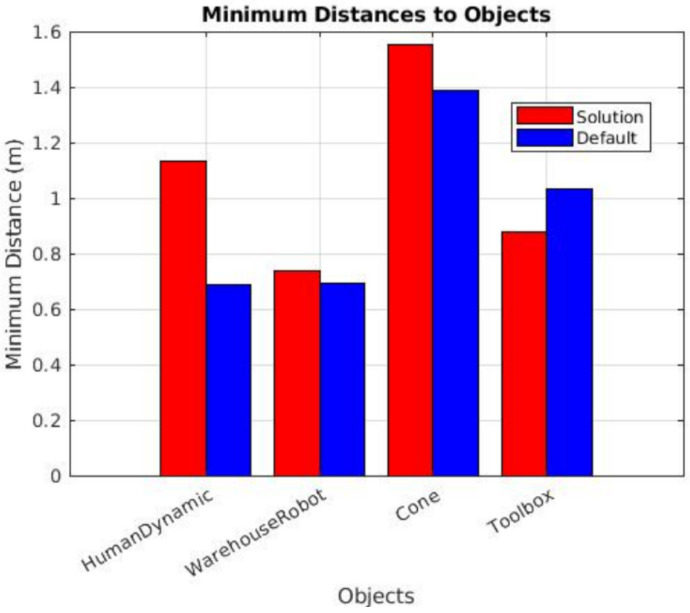
Estimated minimum distances from the robot to the moving human obstacle. Data was acquired during dynamic tests by analyzing the point of maximum directional change.

In the dynamic obstacle tests (results shown in Figure 19), a moving obstacle (a human) was introduced when the robot entered a specific area. However, due to variations in the robot’s speed across tests, the time taken to approach the human after the trigger point differed, resulting in inconsistent and random relative positions between the robot and the obstacle. Since the human obstacle lacked an odometry sensor and was not in a fixed position, directly tracking the minimum distance between the robot and the obstacle proved to be nearly impossible. To address this challenge, an alternative approach was adopted: the minimum distance was estimated by identifying a coordinate where both the solution and default tests exhibited the most significant change in direction along the robot’s path. This change in direction is assumed to correspond to the moment when the robot detects the moving human, brakes, and alters its trajectory. While this method introduced some noise into the results, it did not significantly impact the overall evaluation of the system’s performance, as no collisions occurred in any of the tests.

The results revealed a substantial difference between the distances maintained by our solution and those of the default navigation stack. Our solution consistently demonstrated significantly larger safety margins, which not only validates the effectiveness of our approach but also highlights its prioritization of safety in hazardous situations involving moving humans in the robot’s autonomous path. This improvement in maintaining safe distances shows the system’s ability to handle dynamic and unpredictable environments with a higher degree of safety.

**Figure 20:**
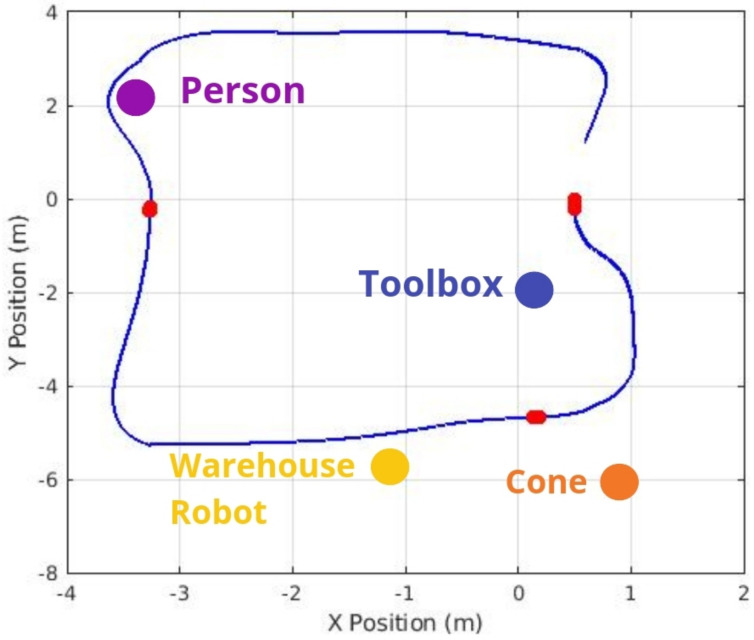
Path of the robot during static tests (blue). The highest brake intensity (marked in red) happens when a large gap between the standard velocity value and the object detection solution with fuzzy logic output is detected.

**Figure 21:**
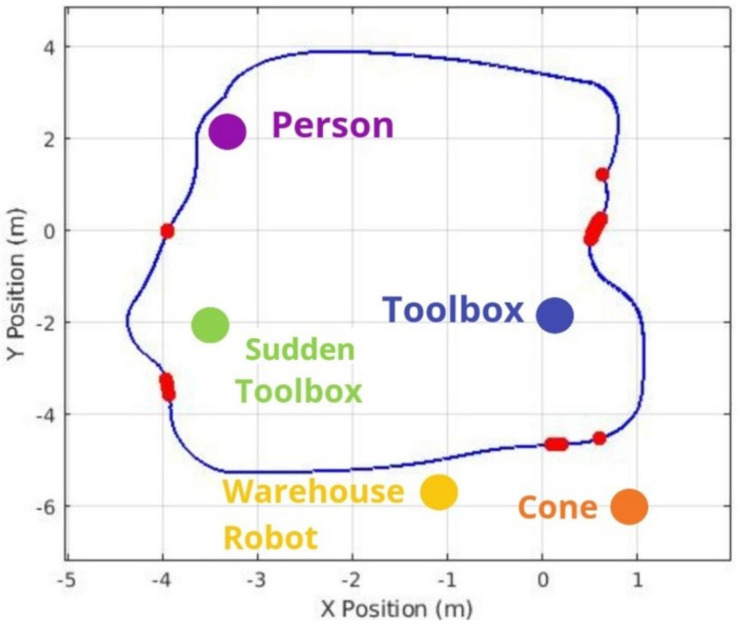
Second braking intensity plot now applied to the sudden obstacle results, adding the sudden toolbox location.

**Figure 22:**
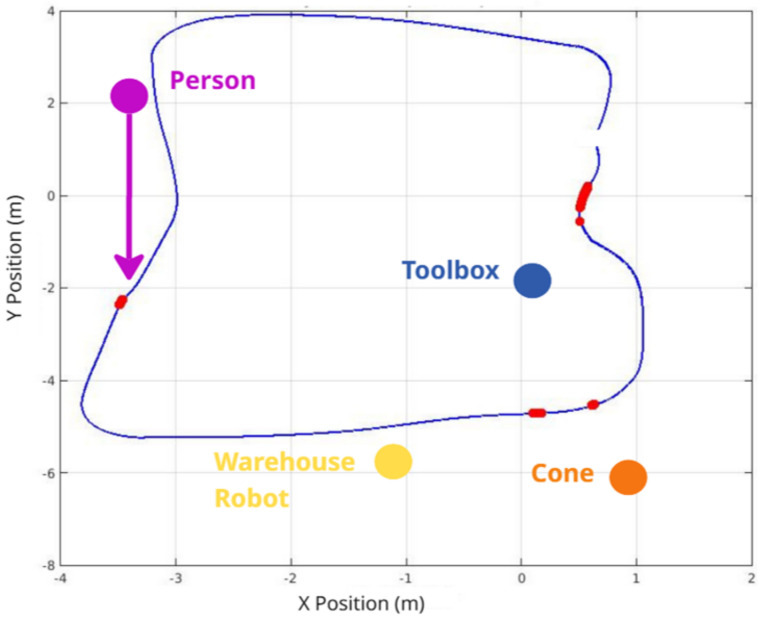
Braking intensity plot results for a dynamic obstacle environment, in which the person moves in the negative Y-axis direction, creating a notably difficult test that highlights the robustness of the solution in the simulation.

Figure 20, Figure 21 and Figure 22 illustrate the most significant differences in braking behavior between the default and proposed solutions applied in static, sudden obstacle appearance, and dynamic tests. The default navigation path is shown in blue, and the red marks illustrate the points where the highest braking intensity was observed. Meanwhile, the purple arrow indicates the moving direction of the dynamic person obstacle in the dynamic tests. These figures demonstrate that our integrated system finds the best braking point based on object detection, especially as the robot approaches obstacles. Notably, there is a marked difference when the robot is approaching a human being, particularly in the dynamic test, as even at minimal proximity to a person (i.e., the closest detectable distance), the system still engaged in meaningful braking. This underscores the crucial role of object detection in the robot’s speed controller and overall safety.

While our solution applied specifically in warehouse robot detection exhibited slightly lower performed than the default technology in certain tests, particularly in scenarios involving human approaches, these results can be attributed to the limitations of the sensor setup. The depth camera, mounted at the base of the Kuka Youbot, encounters challenges when navigating curves, as detection occurs immediately after a turn. Furthermore, the camera’s height limits its field of view, often cropping the legs of a tall person, which does not allow the algorithm to detect the full body. Consequently, these constraints prevented the system from improving performance during all tests and scenarios.

## 5. Conclusions and Future Work

This work proposed an autonomous obstacle-avoiding mobile robot designed for human–robot collaborative environments. It integrated a fuzzy logic module with a YOLOv3-based object detection framework. The proposed approach demonstrated effective navigation and obstacle avoidance in dynamic settings, maintaining safety and efficiency. The simulation tests obtained in this work confirmed that the proposed framework could make the robot dynamically adapt its speed when detecting humans and objects, overcoming safety challenges discussed in the introduction, such as the need for systems that guarantee safety without compromising productivity. In addition, it is important to highlight that the approach demonstrated the ability to operate in dynamic scenarios, which is a demand in the area of collaborative robotics in industry.

Fundamentally, this solution proves how applying advanced techniques like fuzzy logic, even on limited hardware, can enhance human–machine interaction in collaborative environments. Adapting existing technologies to specific hardware, this approach shows that robotic systems can be made more reliable, efficient, adaptable, and safe, making them better suited to real-world collaborative applications when advancing in parallel with the latest researched technologies.

New implementations are foreseen for future work. For instance, although more recent object detection models exist (e.g., YOLOv5, YOLOv8, and Transformer-based architectures), YOLOv3-Tiny was selected in this study for its adequate accuracy and reliable real-time performance. However, we intend to explore the integration of more advanced models in future real-world deployments to potentially enhance detection robustness and generalization. Another important future improvement includes the integration of 3D object detection techniques to better estimate object shapes and volumes. Although the current system uses depth data and PointCloud2-based costmaps for 3D-aware navigation, the explicit reconstruction of object geometries through 3D bounding boxes or mesh models may further improve obstacle representation, particularly in complex or cluttered environments. This capability could enhance motion planning, especially in cases where object shape influences clearance and safety margins. The authors will focus on enhancing the robot’s perception capabilities with additional sensors like LiDAR and expanding testing to real-world industrial environments to validate the system’s scalability and performance under varied conditions. Additionally, future work will include more comprehensive experiments by incorporating complex scenarios with additional obstacles, barriers, and dynamic elements, such as moving persons. These enhancements aim to ensure more realistic evaluations and further refine the system’s adaptability and robustness in collaborative industrial settings. Further research will also explore integrating more advanced deep learning algorithms to improve obstacle detection and decision-making in real-time. Finally, future work will include a theoretical framework to model and analyze the relationships between control inputs, system dynamics, and environmental uncertainties.

## Figures and Tables

**Table 1:** Related works.

Approach	Year	Type of Approach	Adaptability	Computational Complexity	Real-Time Applicability	HRC Suitability
Ali et al. [33]	2015	Type-2 Fuzzy for Collision Avoidance	Moderate	Moderate	Low	Low
Moon and Jajulwar. [45]	2016	Fuzzy Logic Controller	Moderate	Low	Moderate	Low
Henke dos Reis et al. [43]	2019	YOLO + RGB-D Perception	Moderate	Moderate	Moderate	Moderate
Dang et al. [27]	2023	CNN + RGB-D Sensing	High	High	High	High
Achirei et al. [46]	2023	CNN + Model Predictive Control	High	High	Moderate	Moderate
Lopez-Velásquez et al. [44]	2024	Deep Learning + Fuzzy Behavior Trees	Very High	High	High	Very High
Proposed Approach	2024	YOLO + RGB-D + Fuzzy Logic	Very High	High	Very High	Very High

**Table 2:** Costmap parameters.

Parameter	Value
footprint	0.21, 0.165
footprint_padding	0.05
observation_sources	points
sensor_frame	camera link
data_type	PointCloud2
topic	/camera/depth/points
min_obstacle_height	0.05
inflation_radius	0.30

**Table 3:** YOLO Parameter Settings.

Optimizer	SGD (learning rate = 0.01)
Epochs	10,000
Batch size	64
Batch subdivisions	16
Classes	4
Filters	27
Image Size	416 × 416
Weight Decay	0.0005

**Table 4:** Fuzzy rules for angular velocity.

Distance/Turn [m]	H Clockwise	Clockwise	L Clockwise	No Turn	L Counter-Clockwise	Counter-Clockwise	H Counter-Clockwise
**0–1**	VH Left Turn	VH Left Turn	VH Left Turn	No Movement	VH Right Turn	VH Right Turn	VH Right Turn
**0.5–2**	H Left Turn	VH Left Turn	VH Left Turn	No Movement	VH Right Turn	VH Right Turn	VH Right Turn
**1.5–3**	M Left Turn	H Left Turn	M Left Turn	No Movement	M Right Turn	M Right Turn	M Right Turn
**2.5–5**	M Left Turn	H Left Turn	M Left Turn	No Movement	M Right Turn	M Right Turn	M Right Turn
**4–6**	M Left Turn	H Left Turn	M Left Turn	No Movement	M Right Turn	M Right Turn	M Right Turn

**Subtitles:** VH = Very High, H = High, L = Low, M = Moderate.

**Table 5:** Acceleration rules for the robot.

Distance/Velocity	Very Slow	Slow	Medium	Fast	Very Fast
0–1 m	zero	−0.1 m/s	−0.2 m/s	−0.4 m/s	−0.4 m/s
0.5–2 m	zero	zero	−0.1 m/s	−0.2 m/s	−0.4 m/s
1.5–3 m	+0.1 m/s	zero	zero	−0.1 m/s	−0.2 m/s
2.5–5 m	+0.1 m/s	+0.1 m/s	zero	zero	−0.1 m/s
4–6 m	+0.1 m/s	+0.1 m/s	+0.1 m/s	zero	−0.1 m/s

**Table 6:** Acceleration multipliers.

Class	Accel Factor	Slowdown Factor
Toolbox	1.7	0.5
Robot	1.5	0.5
Cone	1.0	0.7
Person	0.7	1.0

**Table 7:** Test results for static, sudden, and dynamic environments.

Static Test Results (average best runs from 32 tests)					
**Type**	**Completion** **Rate**	**Speed** **(m/s)**	**Distance** **(m)**	**Path** **Variation** **(m)**	**Collisions**
Default	100%	0.463	25.62	4.57	0
Solution	100%	0.441	25.22	4.51	0
**Largest Speed Difference (m/s)**		0.124			
**Sudden Toolbox Test Results (average best runs from 32 tests)**					
**Type**	**Completion** **Rate**	**Speed** **(m/s)**	**Distance** **(m)**	**Path** **Variation** **(m)**	**Collisions**
Default	100%	0.448	27.31	4.41	0
Solution	100%	0.422	25.87	4.64	0
**Largest Speed Difference (m/s)**		0.182			
**Moving Person Test Results (average best runs from 32 tests)**					
**Type**	**Completion** **Rate**	**Speed** **(m/s)**	**Distance** **(m)**	**Path** **Variation** **(m)**	**Collisions**
Default	100%	0.484	25.23	4.39	0
Solution	100%	0.451	24.86	4.55	0
**Largest Speed Difference (m/s)**		0.207			

## Data Availability

The original contributions presented in this study are included in the article. Further inquiries can be directed to the corresponding author.

## Abbreviations

The following abbreviations are used in this manuscript:

AGV	Autonomous Guided Vehicle
CNN	Convolutional Neural Network
DWA	Dynamic Window Approach
FL	Fuzzy logic
HRC	Human–Robot Collaboration
HRI	Human–Robot Interaction
LIDAR	Light Detection and Ranging
ROS	Robot Operating System
RRC	Robot-Robot Collaboration
SLAM	Self-Locating and Mapping
SITL	Software in the Loop
YOLO	You Only Look Once Version
